# Attitudes toward and preparedness for lung transplantation among individuals with cystic fibrosis in the era of highly effective modulators

**DOI:** 10.1186/s12890-024-03163-x

**Published:** 2024-07-18

**Authors:** Nora Burdis, Siddhartha G. Kapnadak, Lauren E. Bartlett, Oliver McElvaney, Tijana Milinic, Travis Hee Wai, Allison V. Lange, Nick Reid, Jordan M. Dunitz, Joanne L. Billings, Joseph M. Pilewski, Milene Saavedra, Christopher H. Goss, Andrea L. Hartzler, Kathleen J. Ramos

**Affiliations:** 1 Department of Medicine, Division of Pulmonary, Critical Care, and Sleep Medicine, University of Washington, 1959 NE Pacific St, Box 356522, Seattle, WA 98195 USA; 2 Division of Pulmonary Sciences and Critical Care Medicine, Department of Medicine, University of Colorado Anschutz Medical Campus, Aurora, CO USA; 3 Department of Biomedical Informatics and Medical Education, University of Washington, Seattle, WA USA; 4 Division of Pulmonary, Allergy, Critical Care and Sleep Medicine, Department of Medicine, University of Minnesota, Minneapolis, MN USA; 5 Division of Pulmonary, Allergy & Critical Care Medicine, University of Pittsburgh Medical Center, Pittsburgh, PA USA; 6 Department of Medicine, National Jewish Health, Denver, CO USA; 7 Seattle Children’s Research Institute, Seattle, WA USA

**Keywords:** Lung Transplantation, Medical Decision Making, Cystic Fibrosis, CF

## Abstract

**Background:**

Outcomes for individuals with cystic fibrosis (CF) have improved due to highly effective modulator therapy (HEMT). However, lung transplant (LTx) remains an important treatment for people with advanced lung disease. This study assessed attitudes and knowledge about LTx in the HEMT era.

**Methods:**

All patients from the University of Washington CF clinic were surveyed March 25-May 30, 2020. Questions addressed self-rated LTx preparedness and knowledge, as well as barriers and facilitators to discussing LTx. Demographic and clinical data were extracted from the electronic health record.

**Results:**

There were 159/224 (71%) responses. Respondents had a median forced expiratory volume in one second (FEV_1_) of 70%, and 142 (89%) were on modulatory therapy. One hundred thirteen (71%) respondents felt that it was moderately or very important to be prepared to make decisions about LTx, though only 56 (35%) felt moderately or very prepared. Only 83 (30%) and 47 (52%) participants correctly answered questions about life expectancy and improved quality of life after LTx, respectively. Respondents with Medicaid insurance less frequently answered questions correctly. The most common barriers to discussing LTx were fear of being a burden on loved ones for 58 respondents (36%) and cost of LTx for 46 (29%). Most participants (94%) trusted their CF doctor, and 75% of participants selected trust as a facilitator for LTx discussions.

**Conclusions:**

Many individuals with CF, especially those with lower socioeconomic status, lacked knowledge and did not feel very prepared for decisions about LTx. Earlier education and discussions about LTx represent an area for improvement in CF care.

**Supplementary Information:**

The online version contains supplementary material available at 10.1186/s12890-024-03163-x.

## Background

The 2019 Cystic Fibrosis Foundation (CFF) lung transplant (LTx) referral guidelines recommend early discussion of LTx in the framework of an individual’s disease trajectory for all people living with cystic fibrosis (CF), and annually as a potential treatment option once the forced expiratory volume in one second (FEV_1_) falls to less than 50% of predicted [1]. While LTx is not the right treatment option for all people with advanced CF lung disease, early discussion of LTx is useful for all individuals with CF for several reasons. In addition to promoting timely transplant referral for those in need, earlier introduction of LTx can increase the chance that an individual will be a candidate by informing them of possible modifiable barriers to LTx and giving them an opportunity to address those barriers, some of which may take years to modify [1]. Further, regular discussion of LTx may relieve patients’ unstated anxiety by informing them that they do not require LTx. Early discussion of LTx may ease the referral process, and when the discussion is delayed until the patient’s health deteriorates it can be associated with fear, denial, and avoidance of important care elements [2]. Lastly, individuals with CF have identified discussions of prognosis and LTx as important gaps in their CF care [3, 4].

With the introduction of highly effective CF transmembrane conductance regulator (CFTR) modulator treatments (HEMT), individuals with CF are likely to live longer, healthier lives [5, 6]. Despite this, some individuals with CF will likely still need LTx as a life-extending therapy because of ineligibility or intolerance of HEMT, lung disease progression despite HEMT, or pre-existing advanced CF lung disease at the time when HEMT was initiated. For example, 10–15% of the CF population was ineligible for HEMT in 2022 due to age or CFTR mutation [7], and 25% of adults with CF in the US in 2021 had an FEV_1_ < 50% predicted [8].

With these considerations in mind, timely introduction and discussion of LTx in CF clinic remains important, and identifying facilitators and barriers to these conversations is an unmet need. The objective of this study was to determine attitudes toward LTx among individuals with CF in the era of HEMT. Adults across the CF lung disease spectrum were surveyed to delineate knowledge and preparedness for LTx, along with experiences with and attitudes toward LTx discussions in the CF clinic. We hypothesized that individuals with CF would continue to value discussions about LTx despite the widespread availability of HEMT.

## Methods

Investigators at the University of Washington (UW) developed a survey (Supplement, eTable 1) that addressed whether participants had engaged in prior conversations about LTx, current preparedness for decision-making and knowledge about LTx, barriers and facilitators to LTx discussions, and individuals’ expected impact of HEMT on the need for LTx. Additionally, we described features of a potential decision support tool for LTx and asked for feedback on the use and features of such a tool. The survey questions were developed based on clinical experience and prior research related to communication about LTx for people with CF [4]. Three individuals with CF reviewed the draft survey and provided feedback on the language and content prior to its broader dissemination.

All individuals with CF at the UW Adult CF Center were surveyed (Mar 25, 2020-May 30, 2020). Clinical information was obtained from the electronic health record. Respondents provided granular detail pertaining to socioeconomic status.

UW IRB approval was obtained (Study #7475) for survey distribution and the collection of clinical information. An IRB-approved phone script (Supplement, eTable 2) was used to recruit individuals allowing for up to 3 contact attempts. All participants provided verbal informed consent to participate. The survey link was emailed or texted to interested individuals with CF who provided verbal informed consent.

Knowledge regarding timing of LTx, barriers to LTx, and LTx outcomes was assessed by presenting four true statements to participants:

1. Timing: “Lung function (FEV1 % predicted) is an important component of the decision for a CF doctor to recommend lung transplant as a treatment option.”
2. Barriers to LTx: “Being moderately-to-severely underweight or being obese can make a person ineligible for LTx”
3. Quality of Life: “Lung transplant can improve quality of life within a few months after transplant for individuals with CF”
4. Life Expectancy: “After lung transplant, international estimates show that half of lung transplant recipients with CF live longer than 10 years and half of recipients live less than 10 years.”

For each statement, participants were asked whether they strongly agreed, agreed, were neutral, disagreed, strongly disagreed, or didn’t know.

Survey responses are presented with descriptive statistics.

Subgroups of interest included degree of lung function impairment (FEV_1_ < 30%, 30–50%, ≥ 50% predicted), United States Medicaid insurance status, highest education level (high school or less, some college, graduate education), CFTR modulator status, self-reported preparedness for LTx decision-making, and whether the respondent had undergone prior conversation about LTx with their doctor. Medicaid insurance is a public health insurance program in the United States available to individuals with incomes at or below 133–138% of the federal poverty level. Statistical analysis was performed using Mann–Whitney-Wilcoxon, Chi-squared, and Fisher’s exact testing depending on sample size.

## Results

During the study period, 224 adults with CF at the UW CF Clinic received the survey, and 159 (71%) completed it. Respondents had a median FEV_1_ of 70% predicted and 9 (6%) had FEV_1_ < 30%, 142 (89%) were on CFTR modulators (132, 83% on HEMT), and 20 (13%) had Medicaid insurance (Table 1). Compared to those who did participate, non-respondents had worse lung function (median FEV_1_ of 56% predicted *p* = 0.01, 9 (14%) had FEV_1_ < 30%, *p* > 0.05) and were more likely to have Medicaid insurance (16, 25% with Medicaid *p* = 0.043).(Supplement, eTable 3).

**Table 1:** Patient demographics for individuals with cystic fibrosis who completed the survey about lung transplant (*n* = 159)

Age (years)	Median (IQR)	32 (29–42)
Gender	Woman	87 (55%)
Insurance	Private	108 (68%)
	Medicaid	20 (13%)
	Medicare	24 (15%)
BMI (kg/m^2^)	Median (IQR)	23.3 (21.1–26.7)
FEV_1_% Predicted	Median (IQR)	70 (51, 87)
	FEV_1_ ≤ 30%, *n* (%)	9 (6%)
	30% < FEV_1_ ≤ 50%	30 (19%)
	FEV_1_ > 50%	119 (75%)
F508del CFTR mutation	No F508del, *n* (%)	13 (8%)
	One F508del, *n* (%)	75 (47%)
	Two F508del, *n* (%)	61 (38%)
	Missing, *n* (%)	10 (6%)
Type of CFTR Modulator	Ivacaftor	14 (9%)
	Lumacaftor/ivacaftor	1 (1%)
	Tezacaftor/ivacaftor	9 (6%)
	Elexecaftor/tezacaftor/ivacaftor	118 (74%)
	Not Used	17 (11%)
Education	Some High School	7 (4%)
	High School or GED	16 (10%)
	Some College	50 (31%)
	College	54 (34%)
	Graduate	32 (20%)
Race	White	150 (94%)
	Black	0 (%)
	Asian	1 (1%)
	Native American	2 (1%)
	Pacific Islander	2 (1%)
	Other	4 (3%)
Ethnicity	Hispanic	4 (3%)
Marital Status	Single	62 (39%)
	Married	90 (57%)
	Divorced	6 (4%)
	Widowed	1 (1%)
Income	No Income	11 (7%)
	< $12.5 k	8 (5%)
	12.5 k to 25 k	10 (6%)
	25 k to 50 k	18 (12%)
	> 50 k	109 (70%)

### Frequency of lung transplant discussions

Sixty-five (41%) respondents had previously discussed LTx with their CF doctor. Notably, 9 (100%) respondents with FEV_1_ < 30% and 24 (80%) with FEV_1_ 30–50% had already discussed LTx with their CF doctor. Of the 66 participants who had prior discussions, 47 (72%) found these discussions moderately or very useful. Interestingly, only 14 (15%) individuals with no prior discussion of LTx thought it would be moderately or very useful, while 18 (20%) didn’t know whether a discussion would be useful. Of the 92 respondents who had not previously discussed LTx with their physician, 53 (57%) reported being moderately or very willing to engage in the discussion (Supplement, eFigure 1). Only 46 (29%) of respondents reported having a prior conversation about LTx with a person with CF who had undergone LTx.

### Preparedness for lung transplant decision-making

**Fig. 1:**
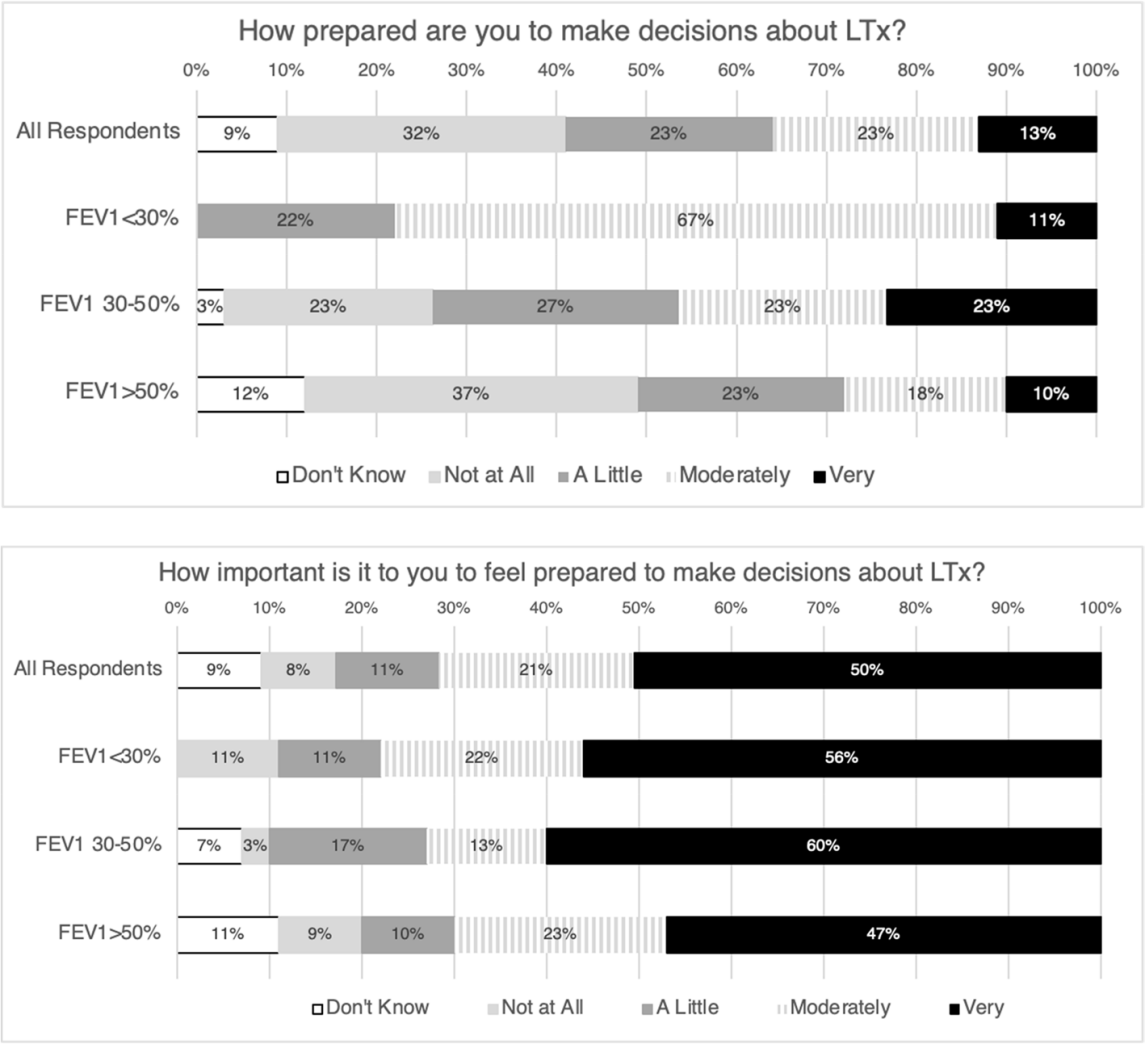
Patient self-reported preparedness (Top) and importance of feeling prepared to make decisions about LTx (Bottom) for the entire cohort and stratified by FEV_1_. The proportion of individuals who reported “Don't know” (white), “Not at all” (light grey), “A little” (dark grey), “Moderately” (hash marks), or “Very” (black) are shown within each FEV_1_ percent predicted stratum (< 30%, 30–50%, > 50%)

Respondents frequently reported feeling unprepared (51, 32%) for LTx decision making or unsure about their preparedness (15, 9%), while few felt very prepared (20, 13%) (Fig. 1). Participants with FEV_1_ < 30% felt more prepared to make decisions about LTx (78% vs. 32%, *p* = 0.01, Fig. 1). Despite the expected difference in self-reported preparedness across the lung disease spectrum, the vast majority of respondents in all FEV_1_ groups acknowledged that it was at least moderately important to be prepared to make decisions about LTx (Fig. 1).

### Knowledge regarding lung transplant

**Fig. 2:**
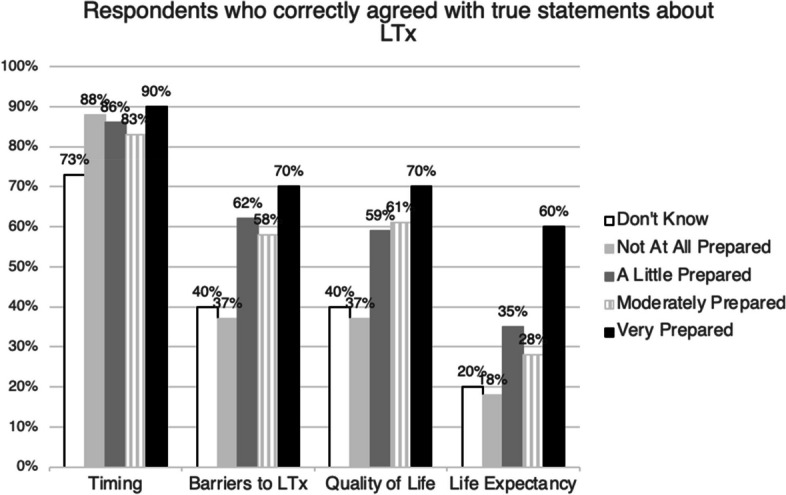
Proportion of respondents who correctly agreed with true statements about LTx, stratified by self-reported preparedness for LTx decision-making. 1) Timing: Lung function (FEV_1_% predicted) is an important component of the decision for a CF doctor to recommend LTx as a treatment option. 2) Barriers to LTx: Being moderately-to-severely underweight or being obese can make a person ineligible for LTx. 3) Quality of Life: LTx can improve quality of life within a few months after transplant for individuals with CF. 4) Life Expectancy: After LTx, international estimates show that half of LTx recipients with CF live longer than 10 years and half of recipients live less than 10 years

Figure 2 displays the proportion of patients who correctly agreed or strongly agreed with each of four true statements, stratified by their self-reported preparedness. Participants who felt very prepared for LTx decisions were generally more likely to answer the knowledge questions correctly, especially about life expectancy (60% vs. 25%, *p* = 0.003). Regardless of self-reported preparedness, 136 (86%) participants knew that FEV_1_ was an important factor in the timing of recommending LTx. The number of participants (47, 30%) who correctly identified the median survival of ten years after LTx for individuals transplanted for CF was relatively lower in all groups compared to other knowledge questions, including only 9 (18%) of those who reported being not at all prepared, and 12 (60%) of those who reported being very prepared correctly agreeing. eFigure 2 shows the proportion of patients who correctly agreed with each statement stratified by lung function. Overall, more advanced lung disease with FEV_1_ < 30% did not clearly correlate with better knowledge about LTx (Timing 77% vs. 86%, Barriers to LTx 33% vs. 54%, Quality of Life 78% vs. 51%, Life Expectancy 44% vs. 29%, *p* > 0.05, Supplement eFigure 2).

### Impact of socioeconomic status and education on preparedness and knowledge

Preparedness for LTx decision-making was compared between participants with Medicaid versus non-Medicaid insurance. Among 20 participants on Medicaid, only 3 (15%) felt moderately or very prepared to make decisions about LTx, and 10 (50%) reported being not at all prepared, the latter of which was nearly double the percentage in non-Medicaid respondents (Supplement eFigure 3). Additionally, respondents with Medicaid insurance performed worse in correctly agreeing with the LTx knowledge statements compared to those with non-Medicaid insurance: Timing (70% vs 86%, *p* = 0.046), Barriers to LTx (30% vs 55%, *p* > 0.05), Quality of Life (40% vs 54%, *p* > 0.05); Life Expectancy (5% vs 33%, *p* = 0.01). Notably, few respondents had low FEV_1_ in the Medicaid subgroup (3 with FEV_1_ < 30%, 2 with FEV_1_ 30–50%, and 15 with FEV_1_ ≥ 50%). Medicaid insurance status was associated with a self-reported annual household income of $25,000 or less (50% vs 14%, *p* < 0.001).

Self-reported preparedness for LTx decision-making did not vary significantly based on education level with approximately one-third of participants feeling moderately or very prepared in the high school, college, and graduate education groups. Despite similar self-assessment of preparedness, respondents with high school education were less likely to answer the LTx knowledge questions correctly compared to those who had attended college or graduate school, though this finding was only statistically significant for the quality of life question (22% vs. 56%, *p* = 0.003, Supplement eFigure 4).

### Barriers and facilitators to discussing lung transplant in cf clinic

Respondents affirmed several barriers and facilitators to LTx discussions in CF clinic (Table 2). The most common barriers were a worry about being a burden on family and friends after LTx, cost of LTx, and a preference to not think about LTx until they were very sick. The most common facilitators were trust in the recommendations from their CF physician and feeling comfortable with raising the topic of LTx. In total 94% of respondents reported trusting their doctor, and 75% selected trust in their doctor as a facilitator for LTx discussions. Of those who reported trust for their doctor, 19% did not feel that this trust helped facilitate conversations about LTx. Among people with Medicaid insurance or high school education or less, barriers and facilitators to discussions were similar to the entire cohort, except for a lower rate of endorsing trust in the CF doctor (45% vs. 80%, *p* = 0.002, Table 2). Facilitators were similar for respondents with FEV_1_ > 70% who had a prior conversation about LTx compared to other respondents (*p* > 0.05).

**Table 2:** Barriers and facilitators to lung transplant discussions in CF clinic endorsed by survey respondents

Barriers	Entire cohort *N* = 159	Medicaid insurance *N* = 20	High school education or less *N* = 23
I worry that I could be a burden on my friends and family if I got very sick	58 (37%)	5 (25%)	10 (43%)
Lung transplant is too expensive	46 (29%)	4 (21%)	8 (36%)
I would prefer not to think about needing a lung transplant until I am very sick	46 (29%)	4 (20%)	4 (17%)
I don’t know enough about why someone gets referred for transplant	30 (19%)	3 (15%)	5 (22%)
I have been told it is not the right time to ask about transplant	22 (14%)	0	1 (4%)
I know someone with CF who died after a lung transplant	20 (13%)	3 (15%)	1 (4%)
Facilitators	Entire cohort *N* = 159	Medicaid insurance *N* = 20	High school education or less *N* = 23
I trust the advice and recommendations of my doctor	120 (76%)	9 (45%)	14 (61%)
It is important for me to feel prepared for a decision about lung transplant prior to when I am “sick enough” to have a transplant	91 (58%)	8 (40%)	11 (48%)
I feel comfortable raising the topic of lung transplant	88 (56%)	7 (35%)	8 (35%)
I am interested in more information about lung transplant and the evaluation process	58 (37%)	7 (35%)	8 (35%)
I have gotten sick in the last year	57 (36%)	7 (35%)	9 (39%)
I have previously discussed lung transplant with my CF doctor	54 (34%)	5 (25%)	7 (30%)
I know someone with CF who is still alive after receiving a lung transplant	49 (31%)	4 (20%)	5 (22%)
I know someone with CF who died without a lung transplant	31 (20%)	3 (15%)	4 (17%)

### Impact of CFTR modulators

**Fig. 3:**
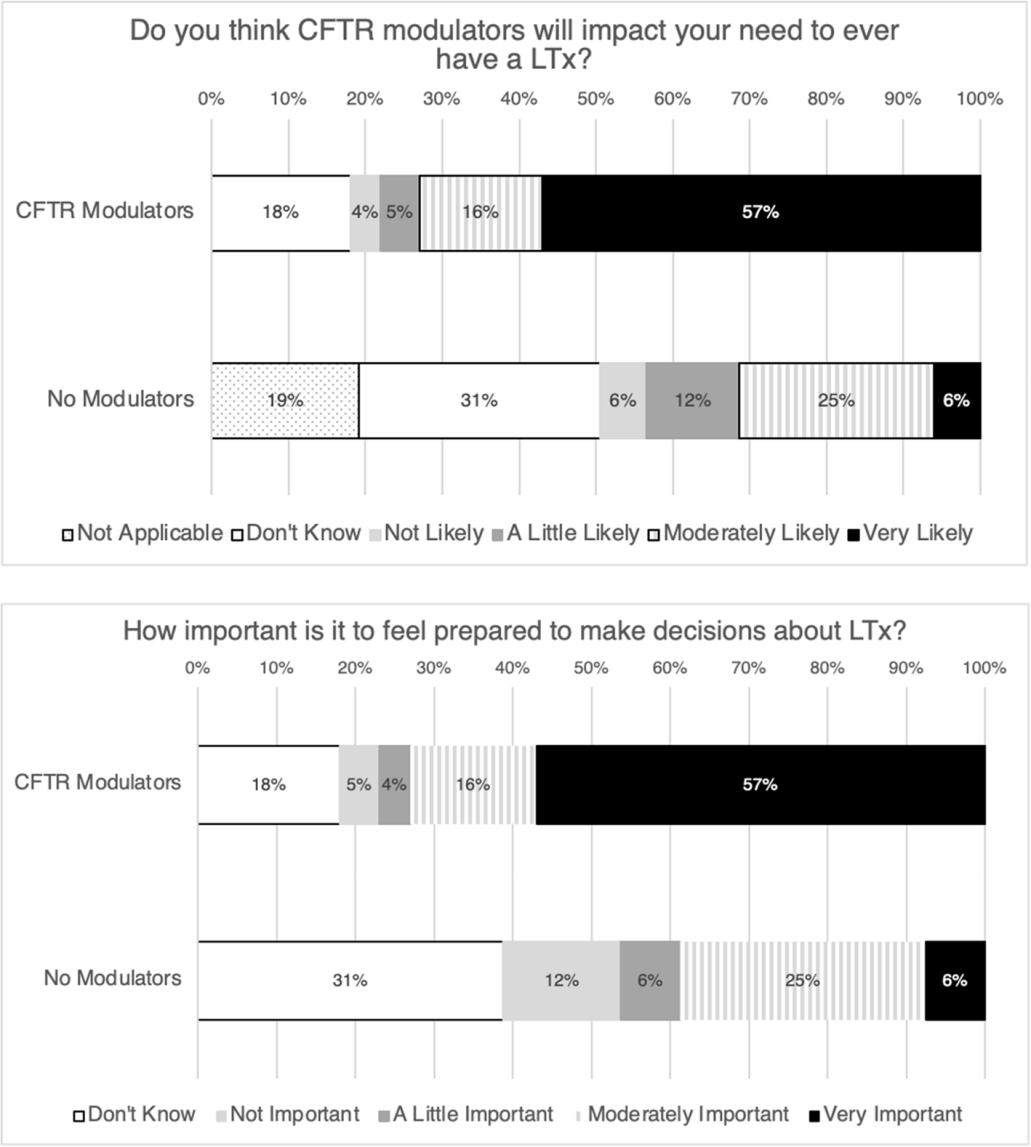
Respondents’ ratings of the likelihood that CFTR modulators will affect their need to ever undergo LTx (Top), and importance respondents place on feeling prepared to make decisions about LTx (Bottom), stratified by whether a respondent is taking a CFTR modulator

Of those taking any CFTR modulator, 81 (57%) responded that it was very likely and 23 (16%) reported it was moderately likely that modulator use would impact their future need to ever undergo LTx (Fig. 3). Despite this, 102 (72%) participants on modulators still reported that it was moderately or very important to be prepared to make decisions about LTx (Fig. 3). As expected, the 17 participants not on modulator therapy expressed more uncertainty regarding the impact of modulators on their future need for LTx.

### Role for a decision support tool for LTx

After reading about shared decision making and the potential role of decision support tools in treatment decisions, 121 (76%) respondents thought a decision support tool for LTx would be at least moderately useful and 121 (76%) would be at least moderately willing to use such a tool once their FEV_1_ is < 50% predicted. One hundred thirteen (75%) respondents reported that an electronic web/computer-based format would be the easiest format to use. Respondents expressed willingness to use the tool alone before a clinic visit (68, 45%), with loved ones before a clinic visit (73, 48%), with their CF physician during clinic (80, 53%), and after discussion with CF doctor (77, 51%). Forty-three (27%) participants were uncertain or at least a little uncomfortable with the idea of the decision support tool containing prognostic information.

## Discussion

In this contemporary cohort of adults with CF, most of whom were taking HEMT, several important conclusions can be made regarding attitudes toward and preparedness for LTx. First, individuals across a wide range of lung function placed a high value on preparedness for LTx decision-making. Though current knowledge about LTx was overall lacking, it was most notable in those with lower socioeconomic status or less educational attainment. Despite the lagging knowledge, consistent with current guidelines a large percentage of those with FEV_1_ < 50% predicted had previously engaged in discussions about LTx with their CF physician. Second, although participants identified the impact that HEMT would potentially have on their ultimate need for LTx, this did not influence the value they placed on preparedness for LTx decisions should the time come. Third, we identified barriers and facilitators to discussing LTx in CF clinic. Worry about being a burden on friends and family was the most commonly reported barrier to discussions, while trust in the advice and recommendations of the CF doctor was the most common facilitator for discussions. Finally, survey respondents reported enthusiasm for an online tool to facilitate learning about LTx once their FEV_1_ is < 50% predicted.

International registry data show a median survival after LTx of approximately ten years for adults with CF, with nearly 25% surviving twenty years [9]. However, despite the ability of LTx to improve quality of life and survival for people with end-staged CF lung disease [10, 11], it has been demonstrated that more individuals with CF and FEV_1_ < 30% die each year than undergo LTx [12]. Deaths without LTx in advanced lung disease represent a potential avenue for improving overall outcomes in CF [1, 13, 14], and opportunities exist for the CF community in relation to timely referral for LTx, early modification of medical or psychosocial contraindications to LTx, and appropriate patient counseling to support informed decision-making regarding LTx [1, 15, 16]. With these concepts in mind, the CFF and recently updated International Society for Heart and Lung Transplantation LTx Candidate selection guidelines emphasize the importance of early introduction of LTx to people with CF well before the need arises to allow more gradual uptake and reinforcement of information that may be challenging to retain [1, 13, 17]. While HEMT will certainly impact the timing and pace of these conversations, providers should recognize the inherent cognitive biases and related potential for delayed introduction of LTx discussions in the HEMT era.

Notably, previous studies demonstrate that patient preference accounts for 25–40% of CF physicians’ decisions to defer LTx referral [15, 16]. To this point, our study demonstrates an important “knowledge gap” in relation to LTx decision-making, where many individuals with CF may actually be lacking the prerequisite knowledge to make informed decisions as they approach LTx. For example, even among the nine respondents with FEV_1_ < 30% predicted in our study, 2 (22%) and 5 (55%) incorrectly answered questions regarding the expected quality of life and survival after LTx, respectively. Moreover, even in the twenty individuals who self-reported to be “very prepared” for LTx decision-making, 2 (10%), 6 (30%), 6 (30%), and 8 (40%) actually answered knowledge questions incorrectly regarding Timing, Barriers to LTx, Quality of Life, and Life Expectancy, respectively, suggesting that in some cases decision-making may be influenced by incorrect but confident assumptions regarding LTx.

Additionally, it is important for providers to recognize that certain situations may require additional attention while educating people with CF about LTx. Deficiencies in preparedness and knowledge appeared most pronounced amongst respondents with lower socioeconomic status and education level, and several socioeconomic issues were identified as barriers to LTx conversations including fear of becoming a burden to caregivers, as well as the cost of LTx. Individuals with CF with Medicaid insurance and those with high school education or less are less likely to be referred or listed for LTx [18, 19]. These situations highlight a special need in certain subpopulations where early planning may be advantageous, not only for financial and caregiver preparation, but also where directed education may be useful. Three LTx decision-support tools in CF have been described with one shown to reduce decisional conflict [20–22], but no applicable tool is publicly available in the HEMT era. A majority of our respondents felt that an online decision-support tool would be useful, and this may represent an opportunity to provide support and education to people with CF in a usable format.

Our study did have limitations. First, although our survey response rate of 71% was robust, it is possible that respondents may be more interested in the topic of LTx than non-respondents and place higher value on LTx preparedness. Non-respondents were more likely to have Medicaid insurance and worse lung function. Only 6% of survey participants had FEV_1_ < 30%. It is possible that our results are skewed by these missed subgroups, but those in the worse lung function cohort are more likely to be engaged in discussions about LTx. Next, assessing LTx knowledge through investigator-designed questions raises the issue of confounding when considering education level, as those with more education may be better at taking tests or may have increased health literacy; also, feeling prepared for a LTx discussion is distinct from having knowledge about LTx. Further, participants’ understanding of the concept of a decision support tool were not formally assessed and participants may have endorsed an interest in a tool without fully understanding it in practice. Additionally, participants had to select barriers and facilitators to discussing LTx from a list and were not able to enter free text responses, so we may have missed barriers or facilitators. Potential barriers to LTx discussions may evolve in the aging CF population on HEMT. Additionally, the results are presented from a cohort of individuals from a single large CF Center, which may limit generalizability. Finally, this survey was conducted during early 2020, so most participants would have been taking elexacaftor/tezacaftor/ivacaftor for < 1 year, and opinions about LTx discussions may have evolved since 2020. It would be useful to repeat this survey over time, especially > 4 years into HEMT and assess how responses evolve with several years of being on HEMT—both in terms of those who continue to feel better and have preserved lung function and those who have started to worsen.

## Conclusions

Although HEMT has and will continue to improve outcomes substantially for individuals with CF, many with advanced lung disease could benefit from LTx, and respondents in our study valued feeling informed and prepared for LTx decision-making regardless of lung function or CFTR modulator status. Many individuals with CF, especially those of lower socioeconomic status, lacked knowledge and did not feel well prepared for decisions about LTx. Earlier education and discussions about LTx represent areas for improvement in CF care.

### Supplementary Information

Supplementary Material 1.

## Data Availability

The datasets used and/or analyzed during the current study are available from the corresponding author on reasonable request.

## Abbreviations

LTx: Lung transplant
HEMT: Highly effective modulator therapy
CF: Cystic fibrosis
CFF: Cystic Fibrosis Foundation
FEV_1_: Forced expiratory volume in one second
CFTR: CF transmembrane conductance regulator
UW: University of Washington
IQR: Interquartile range
