# Supplementary material for: Attitudes toward and preparedness for lung transplantation among individuals with cystic fibrosis in the era of highly effective modulators
Source: BMC Pulm Med. 2024 Jul 18;24:348. doi: 10.1186/s12890-024-03163-x (PMC11256549; doi:10.1186/s12890-024-03163-x)
Supplement: Supplementary file 1 — Supplementary Material 1. [file 12890_2024_3163_MOESM1_ESM.docx]

**Survey-eTable 1**

1. In general, how would you rate your health?
2. Excellent
3. Very Good
4. Good
5. Fair
6. Poor
7. Do you currently take a CFTR modulator (CF specific treatment, based on genotype, for example Trikafta, Kalydeco, Symdeko)?
8. Yes
9. No

If yes, which one:

1. Kalydeco (ivacaftor)
2. Orkambi (lumacaftor/ivacaftor)
3. Symdeko (tezacaftor)
4. Trikafta (elexacaftor/tezacaftor/ivacaftor)

If no, why not: (Please check ALL that apply)

1. Genotype is not eligible for the available modulators
2. Never prescribed
3. Prescribed, but not approved or awaiting approval from insurance
4. Not interested
5. Not effective (previously tried)
6. Significant side effects (previously tried)
7. Other: _____________________
8. Have you already had a conversation about lung transplant with your CF doctor?
9. Yes, I raised the topic first
10. Yes, the doctor raised the topic first
11. Yes, my family member raised the topic in CF clinic
12. Yes, but I do not recall who first raised the topic of transplant
13. No

If no - How useful would this be?

1. Not at all useful
2. A little useful
3. Moderately useful
4. Very useful
5. Don’t know

If no - How willing do you think you would be to participate in this conversation?

1. Not at all willing
2. A little willing
3. Moderately willing
4. Very willing
5. Don’t know

If yes – How useful was this?

1. Not at all useful
2. A little useful
3. Moderately useful
4. Very useful
5. Don’t know

If yes – How willing would you be to discuss transplant again?

1. Not at all willing
2. A little willing
3. Moderately willing
4. Very willing
5. Don’t know
6. Have you already had a conversation (by phone or online) about lung transplant with a person with CF who had a lung transplant?
7. Yes
8. No

If no – How useful would this be?

1. Not at all useful
2. A little useful
3. Moderately useful
4. Very useful
5. Don’t know

If no – How willing do you think you would be to participate in this conversation?

1. Not at all willing
2. A little willing
3. Moderately willing
4. Very willing
5. Don’t know

If yes – How useful was this?

1. Not at all useful
2. A little useful
3. Moderately useful
4. Very useful
5. Don’t know

If yes – How willing would you be to talk to a person with CF who had a lung transplant again?

1. Not at all willing
2. A little willing
3. Moderately willing
4. Very willing
5. Don’t know
6. Do you think the use of CFTR modulators (described above) will have an impact on your need to ever have a lung transplant?
7. Not likely
8. A little bit likely
9. Moderately likely
10. Very likely
11. Not applicable (my genotype is not eligible for the available modulators or I did not tolerate the available modulators)
12. Don’t know
13. Other: ________________________________
14. How prepared do you feel to make decisions about lung transplant as a treatment option?
15. Not prepared
16. A little prepared
17. Moderately prepared
18. Very prepared
19. Don’t know
20. How important is it to you to feel prepared to make decisions about lung transplant as a treatment option?
21. Not important
22. A little important
23. Moderately important
24. Very important
25. Don’t know
26. Do you know anyone (in person or through online communities) who has CF and needs or needed a lung transplant? – (Please check ALL that apply)
27. Being evaluated for transplant
28. Listed for transplant
29. Died without a transplant
30. Decided not to move forward or consider transplant (despite being “sick enough” for transplant”
31. Died after transplant
32. Alive after transplant
33. I don’t know anyone with CF who needs or needed a lung transplant
34. Have you personally considered lung transplant as a treatment option?
35. Does not apply: too healthy
36. Still considering, not yet referred
37. Referred or being evaluated for transplant
38. Listed for transplant
39. Decided not to move forward with or consider transplant (despite being “sick enough” for transplant)
40. Other: _______________________
41. Shared decision making is an approach where clinicians and patients share the best available information when faced with making decisions, and where patients are supported to express their preferences when considering their options.

Decision support tools use print, video, or web-based media to improve knowledge and promote informed decisions about treatment. It can prepare and empower patients and their families for shared decision making with clinicians. A decision support tool can address the risks and benefits of treatments and prepare patients for their role in key decisions.

Using a decision support tool (educational resource) to help you learn about lung transplant, identify your treatment preferences, and guide conversations with your CF doctor once your FEV1 is less than 50% predicted:

1. How useful would this be?
   1. Not at all useful
   2. A little useful
   3. Moderately useful
   4. Very useful
   5. Don’t know
2. How willing do you think you would be to use a decision support tool?
3. Not at all willing
4. A little willing
5. Moderately willing
6. Very willing
7. Don’t know

If response other than “not at all willing” - What would be the easiest way for you to access a decision support tool? (Please check ALL that apply)

- 1. Paper – handout in CF clinic
  2. Electronic – computer/web-based
  3. Electronic – accessible by smartphone as an app
  4. No preference
  5. Other: __________________

If response other than “not at all willing” - How would you prefer to use a decision support tool? (Please check ALL that apply)

1. On my own prior to a CF clinic visit
2. With family or loved one prior to a CF clinic visit
3. With a CF physician during a CF clinic visit
4. After discussion with a CF physician
5. Other: __________________________
6. A risk score can be calculated using information from your medical history to predict the likelihood of someone like you (with your characteristics) to need a lung transplant or die without a lung transplant within 3 years. It can suggest the urgency of the need for evaluation for lung transplant or it can be reassuring if your risk is low.

Including a risk score in the decision support tool that gives you an estimate of your risk of lung transplant or dying without lung transplant within the next three years:

- 1. How comfortable would you be with this?
     1. Not at all comfortable
     2. A little comfortable
     3. Moderately comfortable
     4. Very comfortable
     5. Don’t know
  2. How useful would this be?
     1. Not at all useful
     2. A little useful
     3. Moderately useful
     4. Very useful
     5. Don’t know

Do the following statements make it **more** **difficult to discuss lung transplant** as a treatment option with your CF doctor?

Check YES if the statement is true and this makes it harder to discuss transplant.

Check NO if the statement is true but it does not make it harder to discuss transplant.

Check DOES NOT APPLY if the statement is not true for you.

|  | **YES, makes it harder to discuss** | **No, does not make it harder to discuss** | **DOES NOT APPLY/not true for me** |
| --- | --- | --- | --- |
| 1. I have been told it is not the right time to ask about transplant | □ | □ | □ |
| 1. I don’t know enough about why someone gets referred for transplant | □ | □ | □ |
| 1. I would prefer not to think about needing a lung transplant until I am very sick | □ | □ | □ |
| 1. I know someone with CF who died after a lung transplant | □ | □ | □ |
| 1. I worry that I could be a burden on my friends and family if I got very sick | □ | □ | □ |
| 1. Lung transplant is too expensive | □ | □ | □ |

Do the following statements make it **less difficult to discuss lung transplant** as a treatment option with your CF doctor?

Check YES if the statement is true and this makes it less difficult to discuss transplant.

Check NO if the statement is true but it does not make it less difficult to discuss transplant.

Check DOES NOT APPLY if the statement is not true for you.

|  | **YES, makes it less difficult** | **NO, does not make it less difficult to discuss** | **DOES NOT APPLY/not true for me** |
| --- | --- | --- | --- |
| 1. I am interested in more information about lung transplant and the evaluation process | □ | □ | □ |
| 1. I have gotten sick in the last year | □ | □ | □ |
| 1. I trust the advice and recommendations of my doctor | □ | □ | □ |
| 1. I feel comfortable raising the topic of lung transplant | □ | □ | □ |
| 1. I have previously discussed lung transplant with my CF doctor | □ | □ | □ |
| 1. It is important for me to feel prepared for a decision about lung transplant prior to when I am “sick enough” to have a transplant | □ | □ | □ |
| 1. I know someone with CF who died without a lung transplant | □ | □ | □ |
| 1. I know someone with CF who is still alive after receiving a lung transplant | □ | □ | □ |

Do you agree or disagree with the following statements:

1. Lung function (FEV1 % predicted) is an important component of the decision for a CF doctor to recommend lung transplant as a treatment option.
2. Strongly agree
3. Agree
4. Neutral
5. Disagree
6. Strongly disagree
7. Don’t know
8. Being moderately-to-severely underweight or being obese can make a person ineligible for lung transplant.
9. Strongly agree
10. Agree
11. Neutral
12. Disagree
13. Strongly disagree
14. Don’t know
15. Lung transplant can improve quality of life within a few months after transplant for individuals with CF.
16. Strongly agree
17. Agree
18. Neutral
19. Disagree
20. Strongly disagree
21. Don’t know
22. After lung transplant, international estimates show that half of lung transplant recipients with CF live longer than 10 years and half of recipients live less than 10 years.
23. Strongly agree
24. Agree
25. Neutral
26. Disagree
27. Strongly disagree
28. Don’t know

About you: We would like to ask a few questions about you. These questions help us describe the people who have participated in this study.

What is your current marital status?

1. Single or never married
2. Married or living with a partner
3. Divorced or separated
4. Widowed

What is the highest level of schooling that you have completed?

1. Some high school
2. High school diploma or GED
3. Some college or trade school
4. 4-year college degree (ex. BA or BS)
5. Graduate degree (ex. MA, MS, PhD, MD)

Do you work for income?

1. Yes, full-time (at least 30 hours per week)
2. Yes, part-time (less than 30 hours per week)
3. No
4. Other: ________________

How many people live in your household? _____________

Approximately what was your household income from employment last year?

1. No income
2. Up to $12,500
3. Between $12,500 and $25,000
4. Between $25,000 and $50,000
5. Higher than $50,000

What is your race?

1. Asian
2. Black / African-American
3. Native American / Alaska Native
4. Native Hawaiian / Samoan / Pacific Islander
5. White
6. Other

What is your ethnicity?

1. Hispanic or Latino
2. Not Hispanic or Latino

| eTable 2: The phone script included the following talking points: |
| --- |
| Step 1: Calling the Potential Participant   - Introduction of caller - Brief description of voluntary research study through the University of Washington CF Clinic - Determine whether it is a good time to talk |
| Step 2: Describing the Project   - Ask if they would like to hear more about the study and then provide a more detailed description of the purpose, study procedures, time commitment, and compensation. - Assess interest in participation. - Arrange for email/text of survey link, if interested. - Thank them for their time if they are not interested. |

| eTable 3: Demographics for individuals with CF who completed the survey (n=159) and non-respondents (N = 65) | | | |
| --- | --- | --- | --- |
|  |  | Respondents  N = 159 | Non-respondents  N = 65 |
| Age | Median (IQR) | 32 (29, 42) | 29 (24,38) |
| Gender | Woman | 87 (55%) | 29 (45%) |
| Insurance | Private | 108 (68%) | 39 (60%) |
|  | Medicaid | 20 (13%) | 16 (25%) |
|  | Medicare | 24 (15%) | 10 (15%) |
|  | Other (Tricare, Federal Employee) | 7 (4%) | 0 |
| BMI | Median (IQR) | 23.3 (21.1, 26.7) | 21.4 (19.6, 24.3) |
| FEV_1_ % Predicted | Median (IQR) | 70 (51, 87) | 56 (41,78) |
|  | FEV_1_ ≤ 30%, n (%) | 9 (6%) | 9 (14%) |
|  | 30% < FEV_1_ ≤ 50%, n (%) | 30 (19%) | 15 (23%) |
|  | FEV_1_ > 50%, n (%) | 120 (75%) | 41 (63%) |
| F508del CFTR mutation | No F508del, n (%) | 13 (8%) | 11 (17%) |
|  | One F508del, n (%) | 75 (47%) | 24 (37%) |
|  | Two F508del, n (%) | 61 (38%) | 26 (40%) |
|  | Missing, n (%) | 10 (6%) | 4 (6%) |


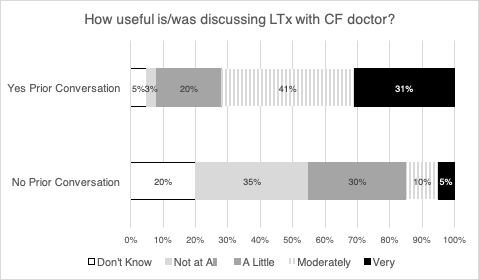


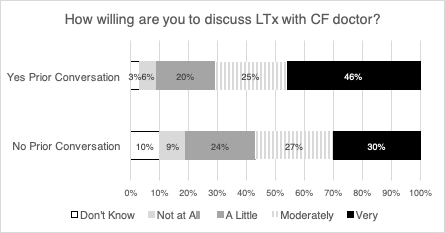


eFigure 1: How useful individuals with cystic fibrosis (CF) thought it would be to discuss lung transplant (LTx) with their CF doctor [Top], and how willing they would be to discuss LTx with their CF doctor [Bottom], stratified by whether they had previously discussed LTx with their CF doctor.


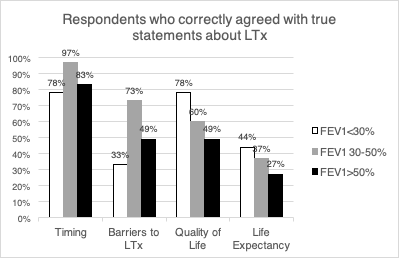


eFigure 2: The proportion of respondents who correctly agreed with true statements about lung transplant, stratified by FEV_1_.

1) Timing: Lung function (FEV_1_ % predicted) is an important component of the decision for a CF doctor to recommend LTx as a treatment option

2) Barriers to LTx: Being moderately-to-severely underweight or being obese can make a person ineligible for LTx

3) Quality of Life: LTx can improve quality of life within a few months after transplant for individuals with CF

4) Life Expectancy: After LTx, international estimates show that half of LTx recipients with CF live longer than 10 years and half of recipients live less than 10 years


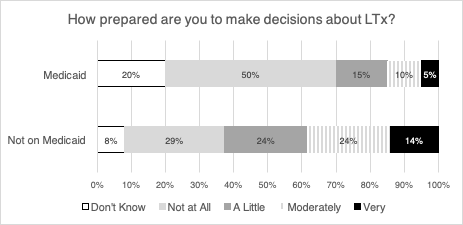


*eFigure 3: Patient self-reported preparedness to make decisions about LTx, stratified by Medicaid insurance status.*


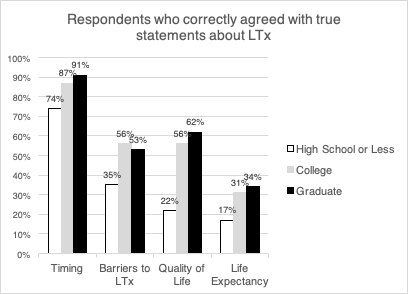


eFigure 4: The proportion of respondents who correctly agreed with true statements about lung transplant, stratified by education level.
